# Crop-Level Screening and Physiological Evaluation Reveal Drought-Protective Effects of Plant-Derived Extracts in Cucumber Seedlings

**DOI:** 10.3390/plants15193055

**Published:** 2026-10-07

**Authors:** Ei Ei, Hyun Hwa Park, Yong In Kuk

**Affiliations:** Department of Oriental Medicine Resources, Sunchon National University, Suncheon 57922, Republic of Korea; eieitza@gmail.com (E.E.); camellia9720@scnu.ac.kr (H.H.P.)

**Keywords:** drought tolerance, plant extracts, oxidative stress, redox homeostasis, antioxidant enzymes, photosystem II, relative water content

## Abstract

Drought stress severely limits crop productivity by impairing water balance, photosynthesis, and oxidative homeostasis. In this study, plant-derived extracts from *Psidium guajava*, *Aloe vera*, *Allium sativum*, and *Medicago sativa* were evaluated as biostimulants for improving drought tolerance in barley, rice, maize, and cucumber seedlings. Crop-level screening showed pronounced interspecific differences, with cucumber exhibiting the highest recovery of shoot fresh weight (79% of the non-stressed control at 0.1% extract concentration, compared to 39% under drought stress alone). Consequently, cucumber seedlings were selected for detailed physiological and biochemical analyses. Under drought stress, extract-treated plants maintained higher relative water content, preserved PSII efficiency (Fv/Fm), and retained chlorophyll and carotenoid contents compared with drought-stressed controls. Drought-induced oxidative damage, reflected by increased hydrogen peroxide and malondialdehyde, was significantly reduced by extract application. These protective effects were associated with higher activities of key antioxidant enzymes and increased accumulation of ascorbate and glutathione. Integrated analyses using oxidative stress indices and z-score heatmaps revealed coordinated improvements across multiple physiological and biochemical parameters, particularly under prolonged stress. Collectively, the results suggest that selected plant extracts enhance drought tolerance by supporting water status, antioxidant capacity, and photosynthetic stability, supporting their potential use as sustainable biostimulants in drought-resilient crop production systems.

## 1. Introduction

Drought is a normal, recurring feature of the climate that occurs in virtually all climatic regimes. Even in more humid climatic zones, drought is often a common feature. Agriculture is one of the key sectors affected by drought. World regions most impacted by drought include South-Central Asia, Southeast of South America, Central Europe and Southeast of the United States [1]. Recently, droughts have led to an economic loss of ~30 billion US dollars globally [2]. Depending on its severity, drought stress can cause severe damage to plants at every stage of their life cycle, from germination to maturity [3], resulting in reduced crop yields [4]. Drought stress can affect the physiological and biochemical characteristics of plant leaves, leading to lower rates of photosynthesis, transpiration, stomatal conductance, and higher proline and carotenoid content [4]. These factors are the main contributing factors to global food shortages [5]. Drought promotes overproduction of reactive oxygen species (ROS) primarily superoxide anion (O2^-^), singlet oxygen (^1^O^2^), hydrogen peroxide (H_2_O_2_), and hydroxyl radical (OH^-^) in cell organelles [6], leading to the initiation of oxidative stress through the peroxidation of cellular membranes [7] and the deterioration of cell structures including proteins, nucleic acids, membrane lipids, and photosynthetic pigments, and activation of programmed cell death [8].

Antioxidant defense machinery protects plants against oxidative stress damage. ROS accumulation induced by various stresses is counteracted by antioxidant enzymes, including superoxide dismutase (SOD), catalase (CAT), ascorbate peroxidase (APX), peroxidase (POD), glutathione reductase (GR), glutathione peroxidase (GPX), guaiacol peroxidase (GOPX), and glutathione-S-transferase (GST), and non-enzymatic low-molecular-weight metabolites including ascorbic acid (AsA), glutathione (GSH), alkaloids, phenolic compounds, nonprotein amino acids, and α-tocopherol, and β-carotene [9]. These enzymes and metabolites not only protect plants from cellular damage, but also fine-tune the concentrations of ROS in order to optimize their functions in metabolism [10].

Irrigation is the first-line solution for agricultural drought, but it is not without its costs and problems (e.g., sustainability and salinity). Strategies to enhance crop drought tolerance beyond irrigation include the use of biostimulants, traditional plant breeding, and genetic modification approaches. A key factor in the successful implementation of these strategies is a better understanding of drought tolerance, which encompasses a series of protective mechanisms that operate at both morphological and physiological levels [11]. Recent regional research has also highlighted the importance of plant adaptation and sustainable vegetation management under environmental stress. For example, studies conducted in the desertification ecosystem surrounding Poyang Lake have demonstrated the potential of drought-adapted vegetation strategies to improve plant establishment and environmental resilience under water-limited conditions [12]. 

The environmentally friendly alternative for enhancing crop yield during drought is the application of plant growth regulators and stress alleviators such as biostimulants [11]. Biostimulants are involved in various processes, including membrane stability, stomatal conductance, osmoprotection, ROS scavenging, nutrient and water use efficiency, and phytohormonal signaling [13], and activate stress-responsive genes through signal transduction and transcriptional regulations [14]. Therefore, the application of biostimulants can alleviate the adverse effects of drought stress and improve plant growth and productivity [14]. Plant biostimulants contain different organic and inorganic substances or microorganisms that can increase plant growth, crop quality, nutrient uptake, and tolerance to biotic and abiotic stresses [15]. Among natural biostimulants, guava, aloe, garlic, and alfalfa extracts are important biostimulants due to the presence of essential mineral nutrients, vitamins, antioxidants, and phytohormones that function to facilitate plant growth and development [16,17].

The beneficial effects of plant extracts on plant growth are well documented [18]. Extracts from guava, aloe, garlic, and alfalfa were selected based on their documented content of phytohormones, antioxidants, and essential nutrients [16,17], which have been shown to promote growth in rice and vegetable crops under optimal conditions. However, their efficacy under drought stress remains largely unexplored. Although certain extracts may improve physiological parameters, their effectiveness on improving productivity and quality under drought stress conditions in protected cereals and vegetables cultivation remains uncertain. Despite evidence that plant extracts can promote growth under optimal conditions, the physiological and biochemical responses they induce under drought stress remain poorly understood, particularly in highly drought-sensitive crops such as cucumber. In the current study, for the first time, the effects of plant extracts on the plant height and shoot fresh weight of barley, rice, maize, and cucumber crops were investigated. In particular, the study aimed to evaluate how the application of plant-derived extracts affects relative water content (RWC), photosynthesis, chlorophyll content, oxidative damage, antioxidant enzymes, and non-antioxidant activity in leaves of cucumber under drought conditions.

The findings support the continued exploration and optimization of biostimulant formulations, potentially leading to more resilient agricultural practices that align with sustainable, environmentally conscious food production systems.

## 2. Results

### 2.1. Crop-Level Screening of Drought Tolerance Under Certain Extract Application

**Figure 1:**
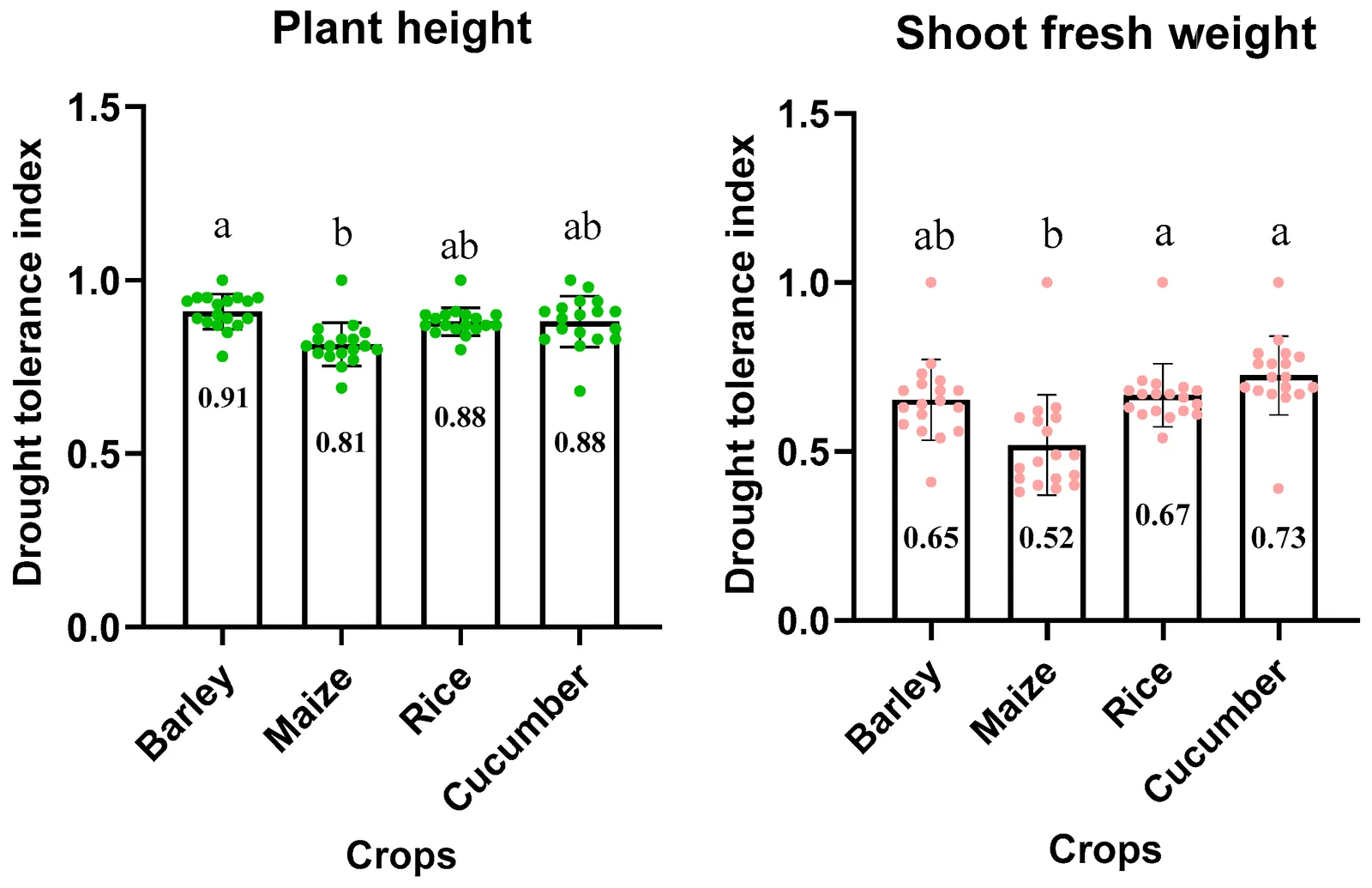
Drought tolerance index of plant height and shoot fresh weight for four crops treated with plant extracts during drought stress. Each dot represents the drought tolerance index (DI) for one extract-concentration combination (4 extracts × 4 concentrations = 16 dots per crop). Bars represent the mean DI averaged across all extract treatments and concentrations (n = 16). Error bars indicate standard error of the mean. Different letters above bars indicate significant differences among crops (Tukey’s HSD, *p* < 0.05).

**Figure 2:**
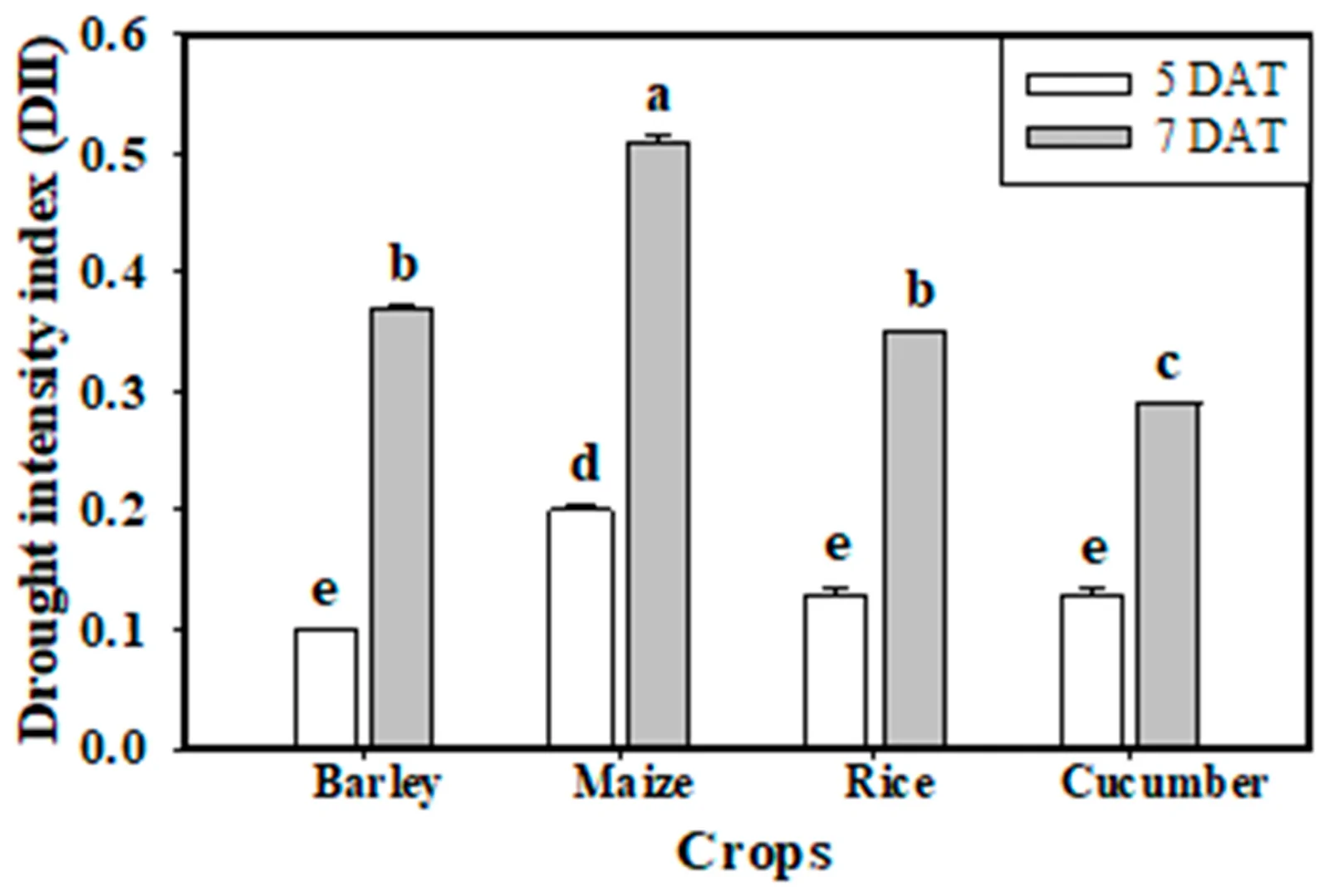
Drought intensity index (DII) of four crops treated with plant extracts under drought stress. Bars indicate the mean DII values averaged over all treatments. Sig nificant differences among crops are indicated by different letters above the bars, based on a one- way ANOVA followed by Tukey’s HSD test (*p* < 0.05). The figure was created using Sigma Plot.

Drought stress reduced plant height and shoot fresh weight (SFW) in all examined crops at the seedling stage; however, tolerance responses differed among crops when averaged across all plant extracts and concentrations at 7 DAT (Figure 1 and Figure 2; Table 1; Table S1. Based on the drought tolerance index (DI), barley showed the highest recovery in plant height (DI = 0.78), whereas maize showed the lowest value (DI = 0.69). For shoot fresh weight, rice exhibited the highest DI value (0.54), followed by maize (0.40) and cucumber (0.39). The drought intensity index (DII) further revealed differences in crop sensitivity to drought stress. Maize exhibited the highest DII values, indicating the greatest susceptibility to water deficit, whereas barley and rice showed intermediate responses. Although cucumber had a relatively low baseline DI for SFW, it exhibited the strongest response to plant extract application, particularly at the 0.1% concentration, with DI increasing from 0.39 under drought stress to 0.79 after treatment. This pronounced recovery, together with cucumber’s relatively high drought sensitivity, makes it a suitable model for investigating the physiological and biochemical responses associated with extract-mediated drought tolerance.

### 2.2. Relationship Between Relative Water Content and PSII Efficiency

**Figure 3:**
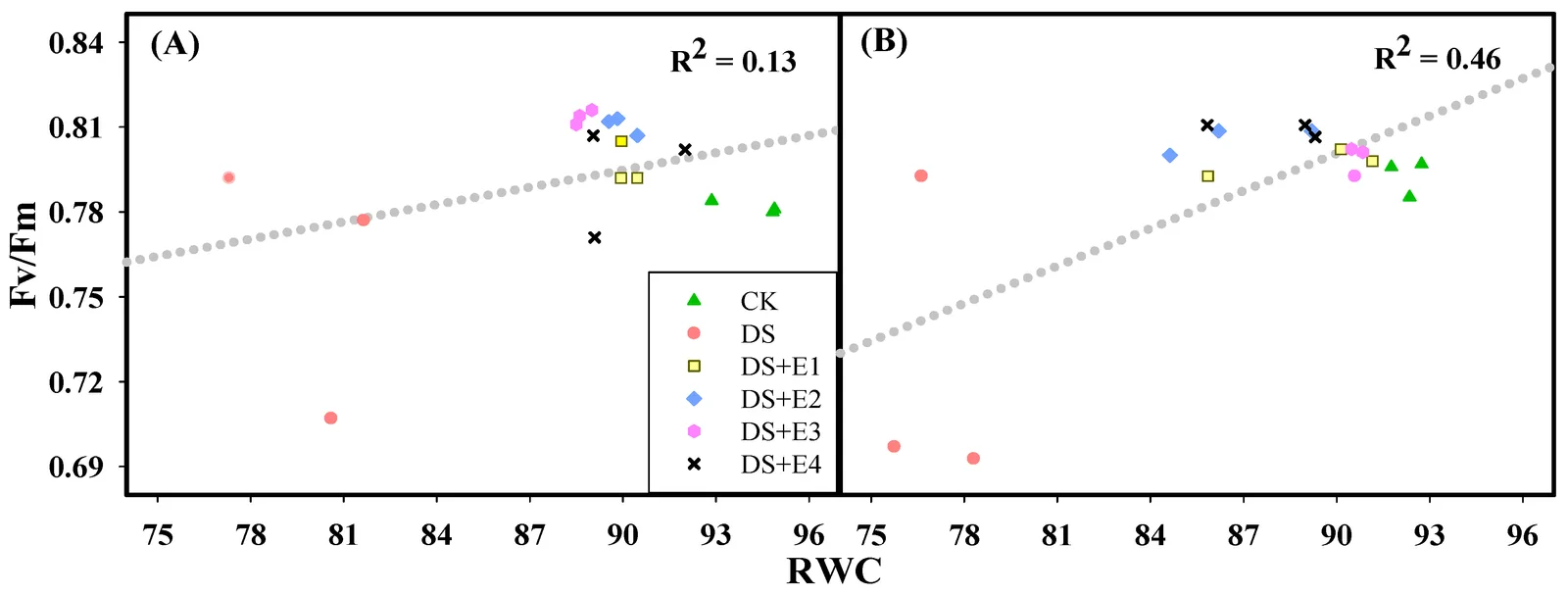
Relationship between relative water content (RWC, %) and maximum quantum efficiency of photosystem II (Fv/Fm) in the seedlings of cucumber leaves at (**A**) 5 and (**B**) 7 DAT under drought conditions following treatment with 0.1% plant extracts. Each point represents a single biological replicate. The scatterplots illustrate the association between leaf water status and photosynthetic performance. Dotted lines indicate linear regression trends, and R^2^ values are shown within each panel. Treatments included control (CK), drought stress (DS), *P. guajava* (E1), *A. vera* (E2), *A. sativum* (E3), and *M. sativa* (E4). The figure was created using Sigma Plot. Data are presented as mean ± SE of three biological replicates (n = 3).

Scatter plots illustrate the association between RWC and Fv/Fm at 5 and 7 DAT (Figure 3). The association between RWC and Fv/Fm was stronger at 7 DAT (R^2^ = 0.46, *p* < 0.05) than at 5 DAT (R^2^ = 0.13, *p* > 0.05), indicating that leaf water status became more closely associated with PSII efficiency under prolonged drought stress. However, the moderate R^2^ value at 7 DAT indicates that RWC accounted for only part of the variation in Fv/Fm, suggesting that additional physiological factors also influenced PSII efficiency. Drought stress reduced both RWC and Fv/Fm at both sampling times. At 5 DAT, drought-stressed plants were distributed at low RWC (~75–78%) and Fv/Fm values (0.70–0.79), whereas extract-treated plants were grouped at higher RWC (~88–92%) with Fv/Fm values ranging from approximately 0.79 to 0.81. At 7 DAT, separation among treatments became more evident, with extract-treated plants clustered at RWC values > 90% and Fv/Fm values comparable to the control (~0.80–0.81), while drought-stressed plants remained at low RWC and Fv/Fm. Overall, higher RWC values were associated with higher Fv/Fm values across treatments and sampling times. 

### 2.3. Total Chlorophyll and Carotenoid Contents

**Figure 4:**
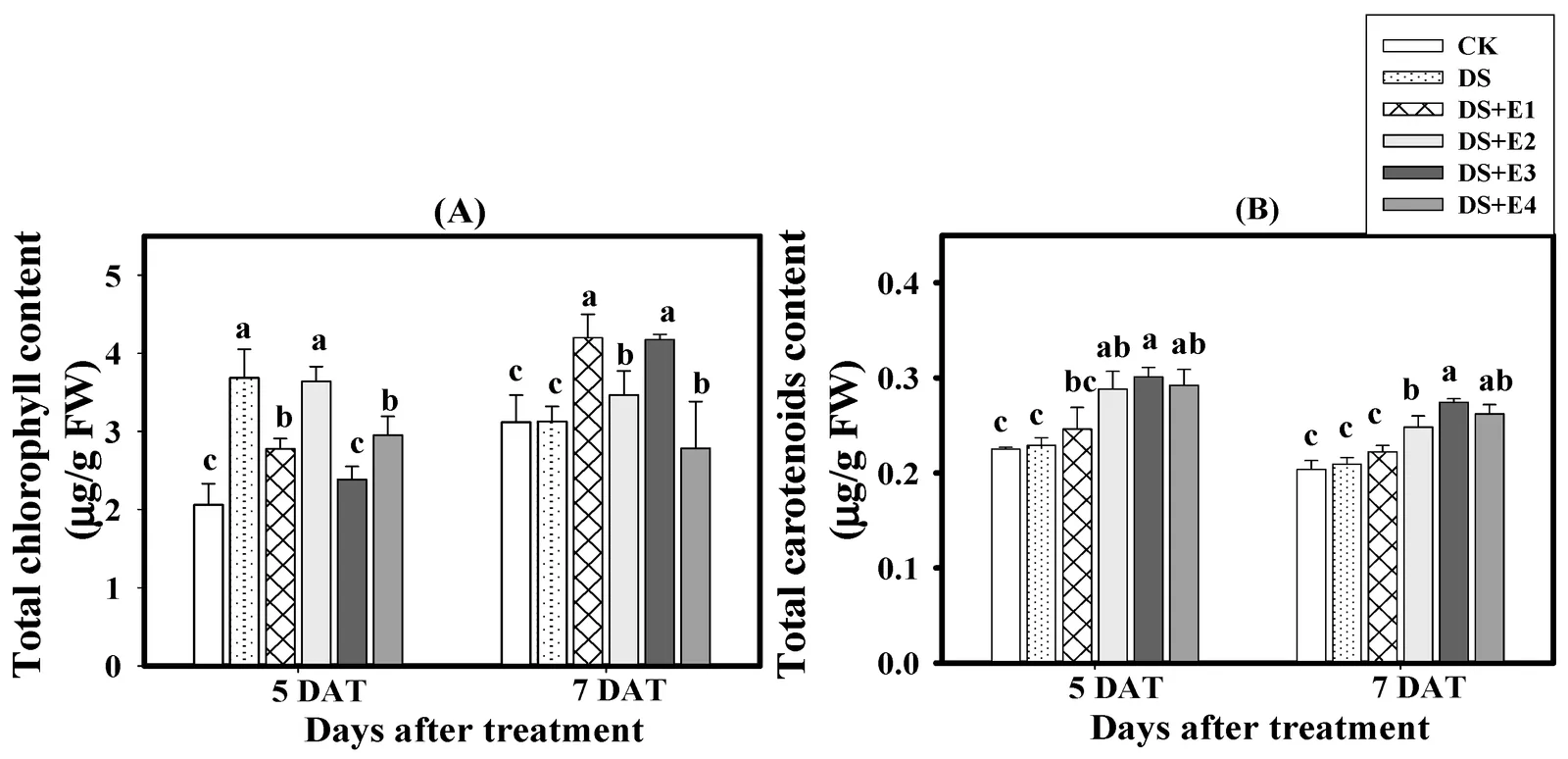
Effects of plant extract treatments on total chlorophyll (**A**) and total carotenoid (**B**) contents in the seedlings of cucumber leaves under drought stress at 5 and 7 DAT. Data from 5 and 7 DAT were analyzed separately using one-way ANOVA followed by the LSD test (*p* < 0.05). Treatments included control (CK), drought stress (DS), *P. guajava* (E1), *A. vera* (E2), *A. sativum* (E3), and *M. sativa* (E4). The figure was created using SigmaPlot. Data are presented as mean ± SE of three biological replicates (n = 3). Different letters above bars indicate significant differences among crops (Tukey’s HSD, *p* < 0.05).

Drought stress affected total chlorophyll and carotenoid contents in the seedlings of cucumber leaves at both 5 and 7 DAT (Figure 4). At 5 DAT, drought-stressed plants exhibited lower chlorophyll contents than the control, whereas extract-treated plants tended to maintain chlorophyll contents comparable to or higher than those of drought-stressed plants. In contrast, carotenoid contents showed relatively small differences among treatments, and no consistent trend associated with extract application was observed. At 7 DAT, differences in chlorophyll contents among treatments became more pronounced, whereas carotenoid contents remained relatively stable and did not exhibit a clear extract-dependent response.

### 2.4. Hydrogen Peroxide and Lipid Peroxidation Accumulation

**Figure 5:**
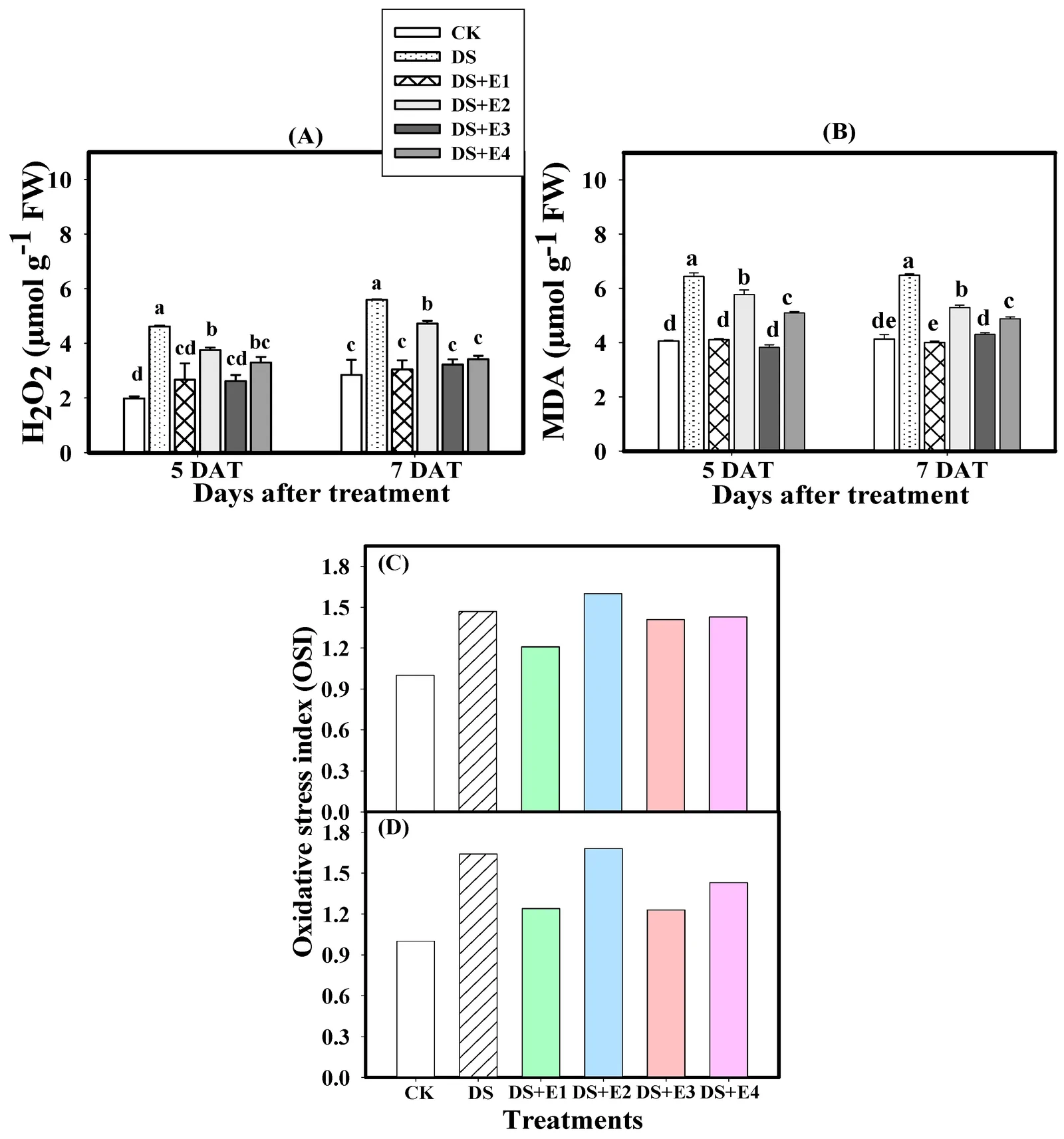
Effects of plant extract treatments on malondialdehyde (MDA) content (**A**), hydrogen peroxide (H_2_O_2_) accumulation (**B**), and oxidative stress index at 5 DAT (**C**) and 7 DAT (**D**) in the seedlings of cucumber leaves under drought stress. Treatments included control (CK), drought stress (DS), *P. guajava* (E1), *A. vera* (E2), *A. sativum* (E3), and *M. sativa* (E4). Data from 5 and 7 DAT were analyzed separately using one-way ANOVA followed by the LSD test (*p* < 0.05). The figure was created using Sigma Plot. Data are presented as mean ± SE of three biological replicates (n = 3). Different letters above bars indicate significant differences among crops (Tukey’s HSD, *p* < 0.05).

Drought stress increased H_2_O_2_ and MDA contents at both 5 and 7 DAT compared to the control, whereas extract-treated plants generally maintained lower levels of both oxidative damage markers (Figure 5). At 7 DAT, H_2_O_2_ and MDA contents remained highest in drought-stressed plants. In contrast, several extract treatments maintained lower oxidative damage indicators than drought-stressed plants. Consistently, extract-treated plants generally showed reduced accumulation of these oxidative damage indicators compared with stress alone. At both sampling times, the responses varied among extract treatments, with E1, E3, and E4 generally maintaining lower oxidative damage indicators than E2. 

In addition, drought stress resulted in higher OSI values than the control at both sampling times. Extract-treated plants showed lower OSI values than drought-stressed plants, with E1 and E3 exhibiting values closer to the control.

### 2.5. Antioxidant Enzyme Responses in the Seedlings of Cucumber Under Drought Stress

**Figure 6:**
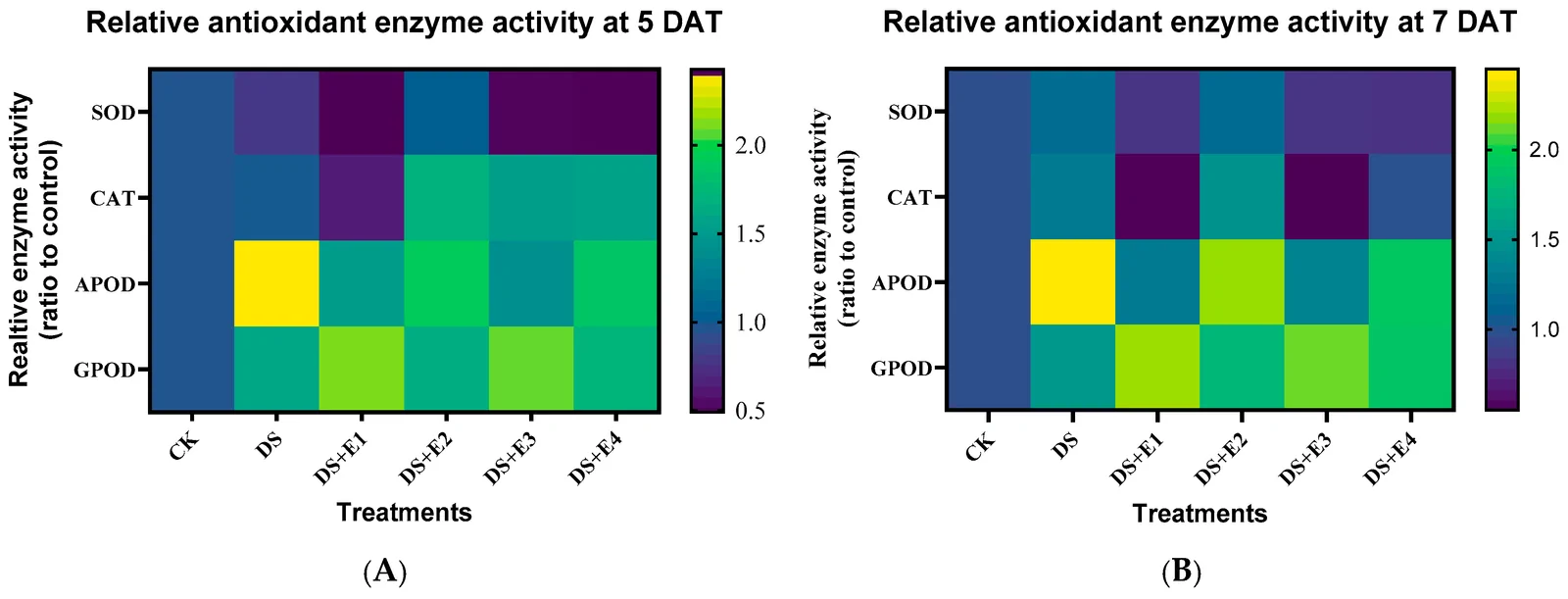
Heatmap analysis of antioxidant enzyme activity in the seedlings of cucumber leaves under drought following plant extract application at 5 and 7 days after treatment ((**A**) and (**B**), respectively). Enzyme activities are expressed as fold-change relative to the non-stressed control (control = 1.0). Each cell represents the mean of three biological replicates. Color intensity indicates the magnitude of change: yellow indicates higher activity; purple indicates lower activity. Values > 1 indicate upregulation relative to control; values < 1 indicate downregulation. Treatments included control (CK), drought stress (DS), *P. guajava* (E1), *A. vera* (E2), *A. sativum* (E3), and *M. sativa* (E4). The figure was generated using GraphPad Prism.

Antioxidant enzyme activities are presented as ratios relative to the control (Figure 6) to facilitate comparison of treatment-induced changes across enzymes with different baseline activities. Absolute activity values (U mg^−1^ protein) are provided in Table S2 to allow assessment of the biological magnitude of changes. Drought stress altered the antioxidant defense system, with enzyme-specific and time-dependent responses. At 5 DAT, CAT, APX, and GPOX activities were generally higher in drought- stressed and extract-treated plants than in the control. SOD activity exhibited comparatively small variation among treatments. Application of plant extracts was associated with higher relative antioxidant enzyme activities, particularly APX and GPOX. Among the extract treatments, E2, E3, and E4 showed stronger CAT, APX, and GPOX responses than E1.

At 7 DAT, differences among treatments became more pronounced. APX and GPOX activities remained relatively high in drought-stressed and extract-treated plants, while CAT activity varied depending on the extract treatment. E2 generally exhibited higher CAT and APX activities than the other extract treatments, whereas E4 showed relatively high GPOX activity. In contrast, SOD activity showed comparatively small changes among treatments at both sampling times. Overall, enzyme activity patterns differed among treatments and sampling times, with APX and GPOX exhibiting greater variation than SOD. 

### 2.6. Non-Enzymatic Antioxidant Responses in the Seedlings of Cucumber Under Drought Stress

**Figure 7:**
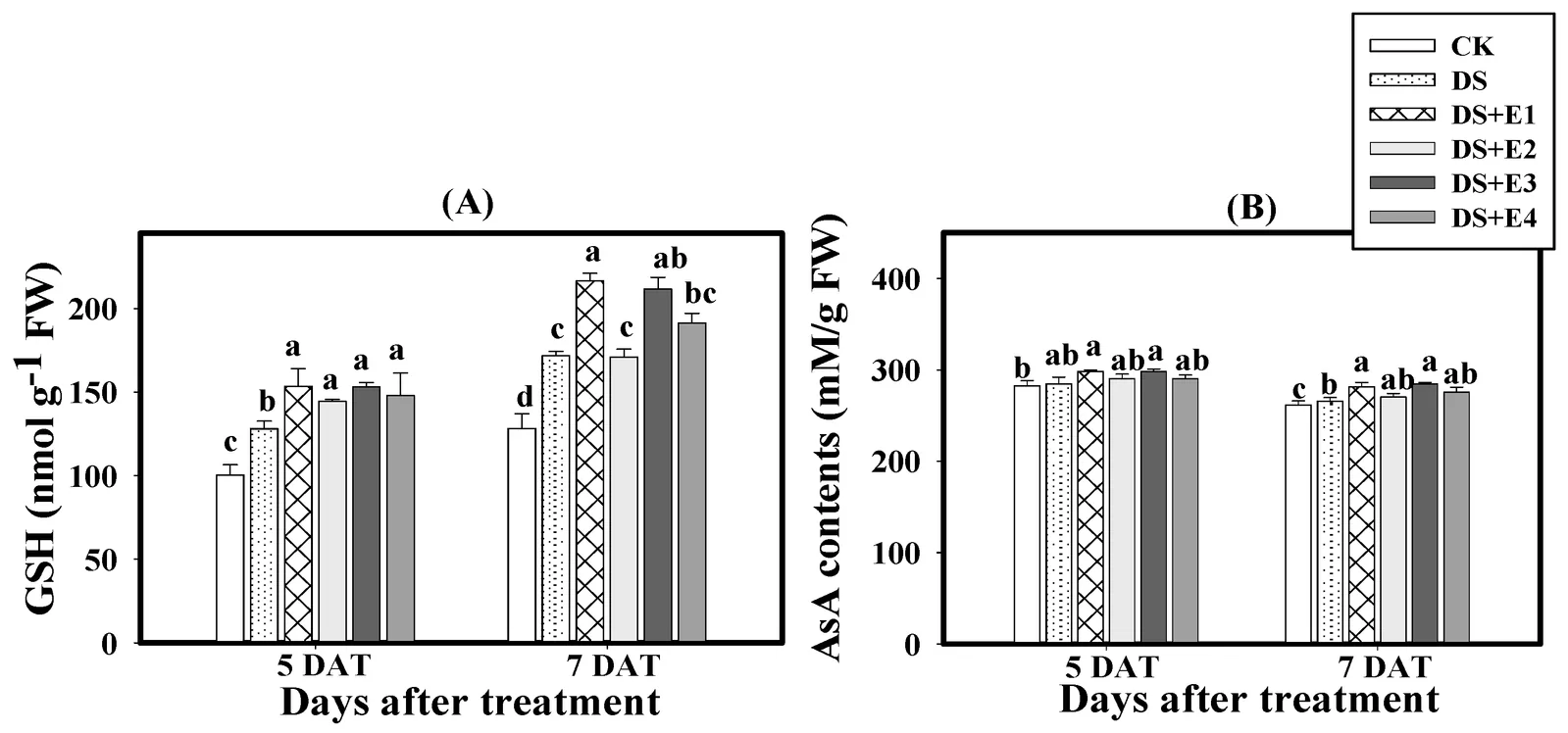
Effects of plant extract treatments on non-enzymatic antioxidant contents in the seedlings of cucumber leaves under drought stress at 5 and 7 DAT. (**A**) Ascorbic acid (AsA) content and (**B**) reduced glutathione (GSH) content. Treatments included control (CK), drought stress (DS), *P. guajava* (E1), *A. vera* (E2), *A. sativum* (E3), and *M. sativa* (E4). Data from 5 and 7 DAT were analyzed separately using one-way ANOVA followed by the LSD test (*p* < 0.05). The figure was created using SigmaPlot. Data are presented as mean ± SE of three biological replicates (n = 3). Different letters above bars indicate significant differences among crops (Tukey’s HSD, *p* < 0.05).

The contents of non-enzymatic antioxidants (AsA and GSH) varied among treatments at both sampling times (Figure 7). At 5 DAT, drought-stressed plants showed lower GSH contents than the extract-treated plants, whereas differences among treatments were limited because all extract-treated plants exhibited comparable GSH contents. For AsA, treatment differences were relatively small, and no consistent extract-dependent pattern was observed. 

At 7 DAT, differences among treatments became more pronounced. Extract-treated plants generally maintained higher GSH and AsA contents than drought-stressed plants. Among the extract treatments, E1 and E3 exhibited high GSH contents, while E4 generally showed intermediate values and E2 remained comparable to drought-stressed plants. Similarly, E1 and E3 exhibited the highest AsA contents, whereas E2 and E4 showed intermediate responses. Overall, extract-treated plants generally maintained higher non-enzymatic antioxidant contents than drought-stressed plants at both sampling times. 

### 2.7. Integrated Physiological and Oxidative Stress Responses and OSI Evaluation

**Figure 8:**
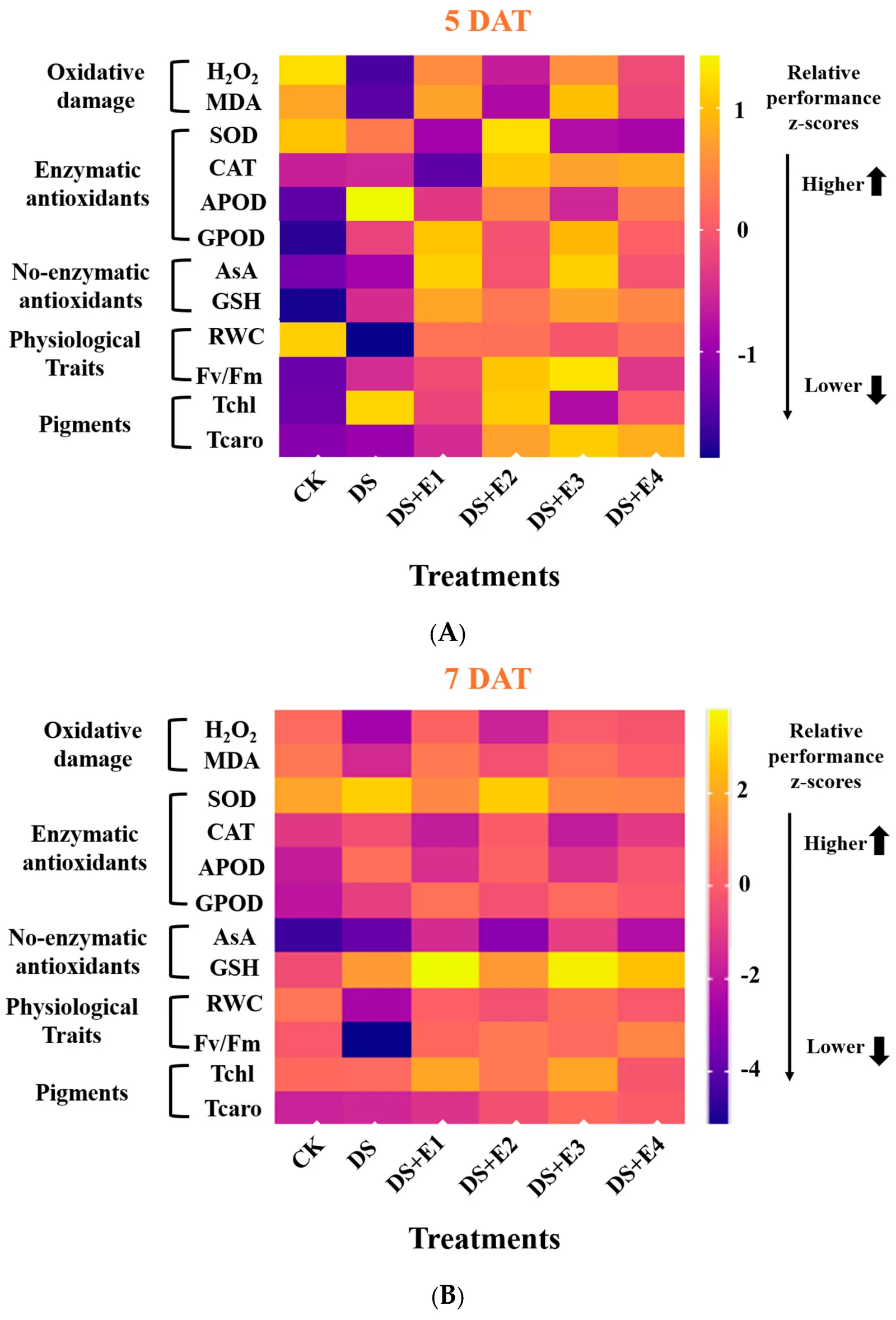
Integrated heatmap analysis of the relative performance (z-scores) of physiological characteristics, photosynthetic pigments, antioxidant defense systems, and oxidative damage markers in the seedlings of cucumber leaves treated with plant extracts under drought stress at five days after treatment (5 DAT) (**A**) and seven days after treatment (7 DAT) (**B**). Data were normalized using z-score transformation to allow comparison of parameters measured on different scales. Z-scores represent the number of standard deviations each value is from the mean: z = (x − µ)/σ. For oxidative damage markers (H_2_O_2_, MDA), scores were inverted (multiplied by −1) so that higher z-scores consistently indicate better stress-mitigation performance across all parameters. Each cell represents the mean z-score of three biological replicates.

Integrated heatmaps (z-score normalized values) provided a system-level overview of the coordinated responses among oxidative damage markers, antioxidant defenses, physiological traits, and photosynthetic pigments at 5 and 7 DAT (Figure 8). Although RWC and Fv/Fm were previously presented separately in Figure 3, they were incorporated into the heatmap to illustrate their integration with antioxidant defenses and oxidative stress-related parameters.

At 5 DAT, the integrated heatmap summarized the patterns previously observed in individual analyses. Drought-stressed plants were characterized by relatively higher H_2_O_2_ and MDA contents and lower values of AsA, GSH, RWC, Fv/Fm, and pigment contents compared with the control. In contrast, extract-treated plants generally exhibited lower oxidative damage indicators and higher values for antioxidant-related and physiological parameters than drought-stressed plants. Differences among extract treatments were observed, with E1 and E3 frequently exhibiting values closer to control than the other extract treatments. 

At 7 DAT, treatment separation remained evident, confirming the coordinated responses previously observed across water status, photosynthetic performance, antioxidant systems, and oxidative damage indicators. Drought-stressed plants continued to exhibit relatively high oxidative damage indicators and lower physiological parameters, whereas extract-treated plants maintained intermediate profiles between the control and drought-stressed plants. Among the extract treatments, E1 and E3 generally showed response patterns closer to the control across multiple parameters. 

OSI values were generally consistent with the integrated heatmap patterns. Drought stress increased OSI relative to the control at both sampling times, whereas extract-treated plants generally exhibited lower OSI values than drought-stressed plants, indicating improved oxidative balance. Among the extract treatments, E1 and E3 generally maintained OSI values closer to the control, suggesting more effective mitigation of drought-induced oxidative stress.

Principal component analysis (PCA) further illustrated the relationships among treatments and measured physiological and biochemical variables (Figure S1). At 5 DAT, PC1 and PC2 explained 36.17% and 27.94% of the total variation, respectively, accounting for 64.11% of the cumulative variation. At 7 DAT, PC1 and PC2 explained 44.50% and 23.06% of the total variation, respectively, accounting for 67.57% of the cumulative variation. Treatment separation became more evident at 7 DAT, with drought-stressed plants associated with higher H_2_O_2_ and MDA, whereas extract-treated plants, particularly *P. guajava* and *A. sativum*, showed distinct response patterns associated with physiological and antioxidant-related variables.

## 3. Discussion

Drought is one of the most detrimental abiotic stresses limiting crop productivity worldwide; however, plant responses to water deficit vary particularly among species and management strategies. The observed interspecific differences in drought tolerance and extract responsiveness likely reflect differences in root architecture, stomatal regulation, and baseline antioxidant capacity. Rice’s stable performance may be attributed to its semi-aquatic evolutionary history, which has selected for constitutive drought avoidance mechanisms, whereas cucumber’s high sensitivity but strong extract responsiveness suggests that its drought vulnerability is primarily physiological rather than structural, making it amenable to biochemical intervention. In contrast, maize displayed limited recovery, particularly in shoot fresh weight, indicating high drought sensitivity and weak responsiveness to extract-mediated mitigation. Notably, cucumber, despite its high drought sensitivity [19], exhibited the greatest overall recovery following extract application, indicating pronounced physiological plasticity under drought stress. Similar extract-mediated drought mitigation has been reported in melon, tomato, and rice, where foliar application of plant extracts enhanced antioxidant capacity, membrane stability, and overall stress tolerance [11,20]. Collectively, these observations support the selection of cucumber as a suitable model for detailed physiological and biochemical analyses. Importantly, the effectiveness of plant extracts was concentration-dependent, with low to moderate doses enhancing stress tolerance, whereas excessive concentrations may impose metabolic constraints, in agreement with previous studies emphasizing the importance of optimized biostimulant application [21,22]. 

The present findings extend our previous studies on the growth-promoting effects of these plant extracts under non-stress conditions [16,17]. In those studies, *P. guajava*, *A. vera*, *A. sativum*, and *M. sativa* extracts promoted plant growth and influenced antioxidant and secondary metabolite responses in rice and vegetable crops under optimal growth conditions. In the present study, these extracts were further evaluated under drought stress, and the results showed that their beneficial effects were retained under water-deficit conditions, particularly at 0.1%. Moreover, whereas our previous studies focused primarily on growth promotion under non-stress conditions, the present study extends these findings by evaluating drought-associated physiological and biochemical responses in cucumber seedlings, including RWC, Fv/Fm, photosynthetic pigments, H_2_O_2_ and MDA accumulation, enzymatic antioxidant activities, and AsA and GSH contents. Thus, the current results provide evidence that the previously observed growth-promoting effects of these plant extracts can also be associated with improved physiological performance and reduced oxidative damage under drought stress.

Improved drought tolerance in extract-treated cucumber seedlings was closely associated with the maintenance of leaf water status and photosynthetic performance. The positive relationship between RWC and Fv/Fm indicates that preservation of tissue hydration is critical for sustaining PSII efficiency under drought stress. Extract-treated plants maintained higher RWC and more stable Fv/Fm values, particularly at 7 DAT. The observed reduction in RWC under drought stress is consistent with disrupted osmotic balance and membrane damage reported in previous studies [23]. In the current study, drought-induced reductions in RWC were accompanied by changes in Fv/Fm, whereas total chlorophyll content did not decrease significantly under drought stress. At 5 DAT, chlorophyll content was numerically higher in drought-stressed plants than in the control, although the difference was not significant, while at 7 DAT, chlorophyll content remained comparable between the drought-stressed and control plants. These findings indicate that the responses of leaf water status and PSII efficiency to drought were not directly accompanied by chlorophyll loss during the experimental period. Similar improvements in leaf water relations and photosynthetic performance following foliar application of seaweed and Moringa leaf extracts have been reported in lettuce, spinach, tomato, and squash under drought stress [11,20,24,25]. 

Drought stress can reduce chlorophyll content through chloroplast damage and enhanced pigment degradation, thereby impairing light-harvesting capacity and photosynthetic performance. However, in the present study, total chlorophyll content did not significantly decrease in drought-stressed plants compared with the control at either sampling time. At 5 DAT, chlorophyll content was numerically higher under drought stress than in the control, although the difference was not significant, while at 7 DAT, the values were comparable. Several extract treatments showed higher chlorophyll contents than the drought-stress treatment, particularly at 7 DAT, suggesting that extract application influenced pigment status despite the absence of a clear drought-induced chlorophyll decline. Carotenoids play an important photoprotective role by dissipating excess excitation energy and scavenging reactive oxygen species generated under stress conditions. Although carotenoid contents did not show a uniform extract-dependent response, several extract treatments maintained or increased carotenoid contents relative to drought stress, particularly at 7 DAT. These results suggest that pigment responses to drought and extract application were treatment- and time-dependent rather than reflecting a uniform drought-induced pigment decline.

Oxidative stress represents a major physiological constraint under drought conditions, as water deficit promotes excessive accumulation of ROS, leading to membrane lipid peroxidation and cellular damage. Elevated levels of H_2_O_2_, MDA indicate intensified oxidative pressure, whereas OSI reflects the accompanying antioxidant enzyme response and should therefore be interpreted together with these oxidative damage markers. Similar reductions in MDA and H_2_O_2_ following biostimulant application under water deficit have been widely reported, supporting the role of biostimulants in protecting membrane integrity and limiting ROS accumulation [26,27]. Notably, the *A. vera* treatment exhibited a higher OSI than the drought-stressed treatment despite the activation of antioxidant enzyme responses. Enhanced antioxidant enzyme activity under stress generally represents activation of cellular ROS-scavenging defenses and contributes to the maintenance of redox homeostasis [28]. This apparently contrasting pattern may reflect the composite nature of the OSI, in which changes in antioxidant-related variables can influence the integrated index and may not directly correspond to the magnitude of oxidative damage. Therefore, the elevated OSI observed with *A. vera* extracts should not be interpreted independently as evidence of greater oxidative injury, but rather together with individual oxidative damage and antioxidant parameters.

The protective effects of the plant extracts may be associated with both direct and indirect modulation of cellular redox homeostasis. Plant-derived extracts contain diverse bioactive compounds that may contribute to ROS scavenging and/or stimulate endogenous antioxidant defense systems. In particular, *P. guajava* leaves are reported to contain diverse antioxidant-associated secondary metabolites, particularly phenolic acids, flavonoids, tannins, and terpenoids [29]. *A. vera* also contains diverse secondary metabolites, including phenolic and flavonoid compounds associated with antioxidant activity [30]. In *A. sativum*, organosulfur compounds constitute a major class of bioactive constituents and, together with flavonoids and other secondary metabolites, have been associated with antioxidant properties [31]. Similarly, *M. sativa* contains bioactive constituents such as saponins, flavonoids, and polysaccharides, and alfalfa saponins have been shown to enhance antioxidant enzyme activity and reduce MDA accumulation under oxidative stress [32]. Collectively, these reported constituents provide a plausible biochemical basis for the lower H_2_O_2_ and MDA accumulation observed in extract-treated cucumber seedlings and may contribute through direct antioxidant activity and/or modulation of endogenous antioxidant defenses. However, the presence and concentration of these particular bioactive compounds cannot be confirmed because the chemical composition of the extracts used in this study was not characterized. As a result, their contribution to the observed drought responses should therefore be viewed as a plausible mechanism rather than a directly established mode of action. 

The antioxidant defense system plays a central role in regulating ROS homeostasis under drought stress. Antioxidant enzymes such as SOD, CAT, and peroxidases function coordinately to detoxify ROS; however, their responses vary depending on species, stress severity, and treatment conditions [33]. In this study, drought stress and extract treatments were associated with increased activities of several antioxidant enzymes, although the magnitude of response varied among enzymes and extract treatments. Previous studies reported that SOD activity decreased in three bentgrass species, with the smallest reduction observed in the drought-tolerant *Agrostis canina* cultivar, whereas SOD activity in sorghum remained almost unchanged under moderate water stress [34], supporting the results of the present study. Under drought stress, CAT activity has been reported to increase in some plant species [35], decrease in others [26,36], or remain relatively stable [37]. 

The APX activity reacted to drought stress and extract applications; however, the extent of the response differed among treatments and sample intervals. APX is an important enzymatic element of the AsA–GSH cycle and contributes to cellular redox regulation as an H2O_2_-scavenging enzyme that uses AsA as an electron donor [38]. Extract application may affect H2O_2_ detoxification and cellular redox regulation during drought stress, according to the observed alterations in APX activity and the preservation of AsA and GSH concentrations. However, the current results should not be construed as proof of regulation of the entire AsA–GSH cycle because other important AsA–GSH cycle enzymes, such as GR, were not assessed.

In contrast, application of *P. guajava* and *A. sativum* extracts resulted in the highest GPOX activity, as well as elevated AsA and GSH contents in cucumber leaves relative to drought stress alone and the control, whereas application of *A. vera* and *M. sativa* extracts resulted in GPOX activities comparable to the drought stress treatment. This response differs from previous reports in spinach [39] and *S. pallescens* Freyn [40], where GPOX was reported to play a comparatively less prominent role in ROS scavenging under drought stress, suggesting th antioxidant responses may vary among species and treatment conditions. AsA and GSH constitute major components of the non-enzymatic antioxidant system and play central roles in cellular redox regulation during abiotic stress. In the present study, drought stress was associated with lower GSH contents, whereas extract-treated plants generally maintained higher GSH contents. The stronger response observed for GSH suggests an important role in maintaining redox balance under drought conditions. AsA contents tended to be higher in extract-treated plants than in drought-stressed plants, although the magnitude of change was less pronounced than that observed for GSH. The maintenance of AsA and GSH, together with the observed APX responses, may contribute to H_2_O_2_ detoxification and cellular redox homeostasis, thereby contributing to the lower oxidative damage observed in extract-treated plants. 

Therefore, increased antioxidant enzyme activities were not interpreted independently as evidence of improved drought tolerance. Rather, the antioxidant responses were evaluated together with the lower H_2_O_2_ and MDA accumulation, maintenance of AsA and GSH, and improved RWC and Fv/Fm observed in extract-treated plants. The combined physiological and biochemical responses suggest improved redox balance and drought-stress mitigation following extract application.

Integrated heatmap analysis provided a system-level perspective on the coordinated physiological and biochemical responses to drought stress and extract application. Drought-stressed plants exhibited a distinct response profile characterized by elevated oxidative damage indicators, altered antioxidant responses, reduced water status and photosynthetic efficiency, and time-dependent changes in pigment contents. In contrast, extract-treated plants showed a more coordinated response pattern, particularly at 7 DAT, characterized by reduced oxidative damage, maintenance of antioxidant defenses, improved physiological performance, and treatment-dependent changes in pigment status, with plants treated with *P. guajava* and *A. sativum* extracts frequently exhibiting response profiles closer to the control. These coordinated responses across multiple traits suggest that plant extracts contribute to integrated drought-stress responses rather than affecting individual parameters independently. These coordinated physiological and biochemical responses may contribute to improved drought tolerance under prolonged stress conditions. Previous studies have shown that exogenous application of compatible solutes significantly improves antioxidant enzyme activities in maize [41], barley [42], cucumber [43], and tomato [44] under drought stress. Similarly, exogenous application of pollen grain extract, containing amino acids, sugars, and essential nutrients, has been shown to increase CAT and SOD activities in *Ocimum basilicum* under drought stress [45]. Foliar application of brown algae extract (*Ascophyllum nodosum*) alleviated drought stress by enhancing antioxidant enzyme synthesis, promoting defense metabolite accumulation, and improving growth and sugar production in sugarcane plants [46]. The PCA further supported these integrated response patterns, with clearer treatment separation observed at 7 DAT than at 5 DAT. At 7 DAT, drought-stressed plants were associated with H_2_O_2_ and MDA, whereas extract-treated plants, particularly those treated with *P. guajava* and *A. sativum*, showed response patterns associated with improved physiological and antioxidant-related traits. Together, the heatmap and PCA indicate that extract effects were reflected across multiple physiological and biochemical responses rather than by changes in individual parameters alone.

Overall, these findings suggest that plant extract applications are associated with improved water relations, photosynthetic performance, and antioxidant responses under drought stress. The results support the use of plant-based biostimulants as environmentally friendly tools for improving crop resilience under drought stress, contributing to sustainable and low-input agricultural production systems. 

Several limitations should be considered when interpreting these findings. First, the study was conducted under controlled greenhouse conditions with a relatively short drought period (7 days), which may not fully represent the complexity and variability of field drought conditions. Second, only a single growth stage was evaluated, and the effectiveness of plant extracts may differ across developmental stages. Third, although the extracts exhibited clear physiological benefits, their biochemical composition and active constituents were not characterized, limiting mechanistic interpretation. Furthermore, while APX, AsA, and GSH were assessed, other key enzymes that are involved in the AsA–GSH cycle, notably GR, were not determined, constraining a thorough evaluation of this antioxidant pathway. Finally, the absence of field validation limits the direct extrapolation of these findings to agricultural production systems. Future research should therefore include multi-location field trials, comprehensive characterization of bioactive compounds, and optimization of application timing, concentration, and frequency to facilitate practical implementation. A limitation of the present study is that soil moisture was not monitored during the drought period. Although the drought treatment was applied using uniform initial hydration and an identical irrigation-withholding period, the actual soil water status experienced by the cucumber seedlings could not be quantitatively determined. Future studies should incorporate soil moisture or soil water potential measurements to more precisely characterize drought intensity and strengthen the interpretation of plant responses to water deficit. 

## 4. Materials and Methods

### 4.1. Plant Materials and Growth Conditions

Rice seeds (*Oryza sativa* L., cv. Hopyeong) were provided by the Jeollanamdo Agricultural Research and Extension Service (Republic of Korea). Seeds of cucumber (*Cucumis sativus* L., cv. Ho Dong Cheong Jang F1), barley (*Hordeum vulgare* L., cv. Saechalssalbori), and maize (*Zea mays* L., cv. Mibaek 2 Ho) were obtained from a commercial seed supplier in South Korea. Plastic pots (16 cm height × 16 cm diameter) were filled with approximately 200 g of commercial horticultural soil (No. 2, Sunghwa, South Korea, consisting of zeolite (5%), perlite (8%), peat moss (8%), vermiculite (12%), cocopeat (67%), and fertilizer (0.4%)) for maize and cucumber, and 130 g for barley. Rice seedlings were grown in nursery pots (6 cm height × 6 cm diameter) containing approximately 130 g of rice nursery soil (No. 1, Sunghwa, South Korea consisting of zeolite (8%), diatomaceous earth (8%), vermiculite (30%), decomposed granite (21.6%), cocopeat (32%) and fertilizer (0.38%)). All plants were maintained in a greenhouse under a 14 h light/10 h dark photoperiod, day/night temperatures of 30 ± 2°C/20 ± 3°C, relative humidity of approximately 70%, and a photosynthetically active radiation (PAR) of 500 μmol m^−2^ s^−1^.

### 4.2. Drought Stress Tolerance and Extract Application in Cereal and Vegetable Crops

A preliminary screening experiment was conducted using the seedlings of maize, cucumber, rice, and barley to compare their responses to drought stress and plant extract applications and to select a crop for subsequent detailed physiological and biochemical analyses. Seedlings at the two–three leaf stage (maize and cucumber) and three–four leaf stage (rice and barley) were placed in plastic containers containing 20 L of tap water for 4 h to ensure uniform hydration. After soaking, all pots were transferred to plastic trays, and drought stress was imposed by withholding irrigation for seven days, during which visible wilting symptoms appeared by day 3–4. Soil moisture was not monitored during the drought period; therefore, the actual soil water status experienced by the plants was not quantitatively determined. To ensure consistent drought treatment among replicates, all pots were uniformly hydrated before drought initiation and maintained under the same greenhouse conditions with an identical irrigation-withholding period. 

The selection of plant extracts, concentration range, and application conditions was based on our previous studies [16,17], in which these extracts were evaluated under non-stress conditions to identify concentrations that promoted crop growth without adverse effects. The present study extended this previous work by evaluating the selected extracts and concentration range under drought-stress conditions. Water extracts were prepared from fresh leaves (guava, aloe, alfalfa) or bulbs (garlic) by homogenizing 100 g of plant material in 1 L of distilled water, followed by filtration through Whatman No. 1 filter paper. Extracts were stored at 4°C and used within 48 h. Detailed preparation protocols are provided in Ei et al. (2024) [16,17]. These extracts were applied as soil drenches (10 mL per pot) at concentrations of 0.05, 0.1, 0.5, and 1%. Extract treatments were applied two days after the initiation of drought stress. To account for the volume of water supplied with the extract treatments, drought-stressed control plants received an equivalent 10 mL soil drench of distilled water at the same time. The application method and volume (soil drench, 10 mL pot^−1^) were adopted from our previous experimental protocol [16,17]. Two control treatments were included: (i) plants maintained under optimal irrigation (non-stress control) and (ii) plants subjected to drought stress and receiving a 10 mL soil drench of distilled water instead of extract (drought-stress control). The detailed treatments included a non-stressed control (CK), a drought-stressed control without extract application (DS), and four drought-stressed treatments receiving the application of *P. guajava* (DS + E1), *A. vera* (DS + E2), *A. sativum* (DS + E3), or *M. sativa* (DS + E4) extract. Extract-only treatments under non-stress conditions were not repeated in the present experiment because the effects of these extracts under non-stress conditions had been evaluated in our previous studies [16,17]; the present experimental design was specifically intended to evaluate their effects under drought stress. At 7 days after treatment (DAT), plant height and shoot fresh weight were measured. Visual damage symptoms were recorded daily throughout the drought period. The experiment consisted of three biological replicates per treatment, with each replicate represented by an independently grown plant. The experiment was repeated twice (two independent runs) under the same experimental conditions.

### 4.3. Drought Stress Tolerance in Cereal and Vegetable Seedlings with Extract Application 

Based on preliminary screening, cucumber seedlings were selected for subsequent detailed physiological and biochemical analyses because they exhibited high drought sensitivity together with a strong recovery response following plant extract application. Shoot fresh weight was given greater consideration than plant height because it provided a more integrated measure of shoot biomass accumulation and showed a clearer treatment response during preliminary screening. The initial concentration range was selected based on our previous studies [16,17], whereas the selection of 0.1% for the detailed drought experiment was based on the results of the present drought-screening experiment. Among the concentrations tested, 0.1% provided consistently favorable recovery of shoot fresh weight across the four crops (Table 1), while higher concentrations (0.5 and 1%) provided no consistent additional benefit. Accordingly, four plant extracts—*Psidium guajava*, *Aloe vera*, *Allium sativum*, and *Medicago sativa*—were applied at 0.1% for subsequent detailed physiological and biochemical analyses in cucumber seedlings. Drought stress was imposed as described above. Leaf samples were collected at 5 and 7 DAT. At each sampling time, one plant per replicate (n = 3) was sampled. Other experimental procedures followed those described in Ei et al. (2024) [16,17].

### 4.4. Measurement of Photosynthetic Efficiency and Pigment Contents

Chlorophyll—*fluorescence* parameter of photosystem II (PSII), the maximum quantum efficiency (Fv/Fm), chlorophyll, and carotenoid contents, were measured in cucumber leaves at 5 and 7 DAT under drought stress. The second fully expanded leaf was used for all measurements.

Chlorophyll fluorescence was measured using a portable pulse-amplitude-modulated fluorometer (PAM-2500, Heinz Walz GmbH, Effeltrich, Germany). Leaves were dark-adapted for 15 min before measurement to ensure full oxidation of PSII reaction centers. Chlorophyll and carotenoid contents were determined following the methods described in Ei et al. (2024) [16,17].

### 4.5. Measurement of Relative Water Content 

RWC was determined in the second fully expanded leaves of cucumber seedlings at 5 and 7 DAT under drought conditions [47]. Empty paper bags were first weighed (W1). Fresh leaf samples were excised, immediately placed into the paper bags, and weighed together with the bags (W2). Samples were then oven-dried at 70 °C for 24 h and weighed again with the paper bags to obtain dry weight (W3). RWC was calculated using the following formula: RWC (%) = [(W2 − W3)/(W2 − W1)] × 100.

### 4.6. Measurement of Lipid Peroxidation and Hydrogen Peroxide Contents

Malondialdehyde (MDA) content was determined using the thiobarbituric acid (TBA) method as described in [48]. Fresh leaf tissue (0.5 g) was homogenized in 25 mL of 0.5% TBA prepared in 20% trichloroacetic acid (TCA). The homogenate was centrifuged at 20,000× *g* for 15 min, and the supernatant was heated at 100 °C for 25 min, followed by rapid cooling. After centrifugation, absorbance was recorded at 440, 532, and 660 nm using a UV spectrophotometer (SPECTROstar Nano, BMG LABTECH GmbH, Ortenberg, Germany). MDA content was calculated using an extinction coefficient of 156 mm^−1^ cm^−1^ following the formula described by Du and Bramlage (1992) [49].

H_2_O_2_ content was measured according to Jana and Choudhuri (1981) [50]. Leaf tissue (0.5 g) was homogenized in 50 mm phosphate buffer (pH 6.8) and centrifuged at 6000× *g* for 25 min. The supernatant was reacted with 0.1% titanium sulfate in 20% (*v*/*v*) sulfuric acid and centrifuged, and the absorbance was measured at 410 nm. H_2_O_2_ concentration was calculated using an extinction coefficient of 0.28 μmol^−1^ cm^−1^.

### 4.7. Measurement of Antioxidant Enzyme Activities and Non-Enzymatic Antioxidants

Antioxidant enzyme activities (SOD, CAT, APX, and GPX) were determined according to the procedures described in Ei et al. (2024) [16]. SOD activity was measured according to Giannopolitis and Ries (1977) [51], with modifications described by Chowdhury and Choudhuri (1985) [52] and Zhang et al. (1995) [53]. The reaction mixture (total volume 300 μL) consisted of 20 μL enzyme extract, nitrogen blue tetrazolium (NBT), 260 mm riboflavin, 260 μM methionine, 2 mm EDTA, and 200 mm phosphate buffer (pH 7.0). The mixture was exposed to light at an intensity of 78 μmol photons m^−2^ s^−1^ for 10 min, and absorbance was measured at 560 nm using a SPECTROstar Nano spectrophotometer (BMG LABTECH GmbH). One unit of SOD activity was defined as the amount of enzyme required to inhibit NBT photoreduction by 50%.

CAT and GPX activities were determined following the methods of Chance and Maehly (1995) [54] and Fu and Huang (2001) [55]. CAT activity was measured by monitoring the decrease in absorbance at 240 nm for 1 min due to H_2_O_2_ decomposition. The reaction mixture (300 μL) contained 150 μL of 200 mm phosphate buffer (pH 7.0), 15 μL of 15 mm H_2_O_2_, 120 μL of distilled water, and 15 μL of enzyme extract. GPX activity was assayed by measuring the increase in absorbance at 470 nm for 1 min, corresponding to guaiacol oxidation. The reaction mixture consisted of 5 μL of 200 mm guaiacol, 280 μL of 200 mm phosphate buffer (pH 7.0), and 15 μL of 40 mm H_2_O_2_. All spectrophotometric measurements were performed using a SPECTROstar Nano spectrophotometer.

APX activity was determined following the method of Chen and Asada (1989) [56] by monitoring the initial rate of ascorbate oxidation. The decrease in absorbance was recorded at 290 nm using a UV spectrophotometer (SPECTROstar Nano, BMG LABTECH GmbH). The reaction mixture (total volume 300 µL) consisted of 150 µL of 200 mm phosphate buffer (pH 7.0), 30 µL of 5 mm ascorbate, 12 µL of 20 mm H_2_O_2_, 30 µL of distilled water, and 15 µL of enzyme extract. The reaction was initiated by the addition of H_2_O_2_, and APX activity was calculated from the linear decrease in absorbance at 290 nm.

AsA content was determined following the method of Law et al. (1983) [57]. Fresh leaf tissue (0.5 g) was homogenized in 5 mL of 5% (*w*/*v*) metaphosphoric acid and centrifuged at 13,000× *g* for 15 min at 4 °C. To quantify total AsA, 100 μL of the extract was mixed with 250 μL of a reaction solution containing 150 mm potassium phosphate buffer (pH 7.4) and 5 mm EDTA, followed by the addition of 50 μL of 10 mm dithiothreitol (DTT). For AsA determination, DTT was replaced with distilled water. After incubation at room temperature for 10 min, 50 μL of 0.5% (*w*/*v*) N-ethylmaleimide (NEM) was added to quench excess DTT. Each reaction mixture was supplemented with 611 μL of a color-developing solution containing 10% trichloroacetic acid, 44% phosphoric acid, 70% ethanol with 4% bipyridine, and 30% FeCl_3_. Samples were incubated at 37 °C for 60 min, and absorbance was measured at 525 nm using a UV spectrophotometer (SPECTROstar Nano, BMG LABTECH GmbH). AsA concentration was calculated using a standard curve prepared with 0–100 μM L-ascorbic acid.

GSH content was determined using an enzymatic cycling assay according to Gaitonde (1967) [58], with minor modifications. Leaf tissue (0.5 g) was homogenized in 6 mL of 5% (*w*/*v*) sulfosalicylic acid and centrifuged at 12,000 rpm for 10 min at 4 °C. For GSH determination, 20 μL of the extract was mixed with 170 μL of distilled water and 700 μL of reaction buffer consisting of 100 mm sodium phosphate buffer (pH 7.5), 6.3 mm EDTA, and 0.248 mg mL^−1^ NADPH. Subsequently, 100 μL of 6 mm 5,5′-dithiobis-(2-nitrobenzoic acid) (DTNB) prepared in reaction buffer was added. The reaction was initiated by the addition of 10 μL of glutathione reductase (0.5 units), and the increase in absorbance was recorded at 412 nm for 1 min using a UV spectrophotometer. GSH concentration was calculated based on the rate of absorbance change and expressed relative to fresh weight.

### 4.8. Statistical and Data Analysis

The experiment followed a completely randomized design with six treatment groups and three replications per treatment. Data collected at 5 and 7 DAT were analyzed separately using one-way ANOVA, with treatment considered as a single factor comprising the non-stressed control (CK), drought-stressed control (DS), and four drought-stressed extract treatments (DS + E1, DS + E2, DS + E3, and DS + E4). Mean separation was performed using the least significant difference (LSD) test at *p* < 0.05. Although repeated measures ANOVA would account for temporal dependency, separate analyses were performed due to differences in sample size and measurement protocols between time points. Heatmap and boxplot visualizations were generated using GraphPad Prism (version 8.0.2). A scatter plot with linear regression trend lines and corresponding R^2^ values, as well as bar graphs, were generated using the SigmaPlot software (version 16). For comparative visualization of biochemical parameters across treatments, data were normalized using a z-score transformation. Z-scores were calculated for each parameter as z = (x−μ)/σ, where *x* is the observed value, *μ* is the mean, and *σ* is the standard deviation of the dataset. The transformed values were used exclusively for heatmap visualization and did not affect statistical analyses. Principal component analysis (PCA) was performed separately for 5 and 7 DAT using standardized (z-score transformed) values of GSH, AsA, SOD, CAT, APX, GPOX, Fv/Fm, total chlorophyll, carotenoids, H_2_O_2_, MDA, and RWC at the biological-replicate level. PCA was used to visualize the relationships between physiological and biochemical variables and the separation of treatment groups.

#### Calculation of Drought Stress and Oxidative Stress Indices

To quantitatively assess drought tolerance and oxidative stress responses, drought- and oxidative-stress-related indices were calculated as derived variables during data analysis.

The drought tolerance index (DI) was calculated according to [59]:

DI = Trait _DS_/Trait _NS_ (1)

where Trait _DS_ and Trait _NS_ represent the mean values of plant height and shoot fresh weight of cucumber under drought stress and non-stress conditions, respectively.

Drought intensity index (DII) was used to evaluate the overall severity of drought stress across crops and was calculated [60]:

DII = 1 − (Ys/Yp)(2)

where Ys is the mean value of plant height and shoot fresh weight of all tested crops under drought stress, and Yp is the corresponding mean under non-stress conditions.

Oxidative stress index (OSI) was calculated as a composite indicator to quantify oxidative stress based on antioxidant enzyme activities [61], as follows:

OSI = ([SOD]/[SOD]0 + [CAT]/[CAT]0 + [APX]/[APX]0 + [GPX]/[GPX]0)/4(3)

where [SOD], [CAT], [APX], and [GPX] represent the activities of superoxide dismutase, catalase, ascorbate peroxidase, and guaiacol peroxidase, respectively, under drought stress with or without extract application, and the subscript “0” denotes the corresponding values in non-stressed control plants. An OSI value greater than 1 indicates elevated oxidative stress, whereas values below 1 indicate reduced oxidative stress relative to the control. Increased antioxidant enzyme activities under drought stress can reflect activation of the enzymatic defense system in response to enhanced ROS production. Therefore, the OSI was used as a composite indicator of the magnitude of antioxidant responses associated with oxidative stress. OSI values were interpreted together with direct oxidative stress markers, including H_2_O_2_ and MDA.

## 5. Conclusions

This study demonstrates that selected plant-derived extracts (*Psidium guajava*, *Aloe vera*, *Allium sativum*, and *Medicago sativa*) have the potential to enhance drought tolerance in crops, with cucumber seedlings exhibiting the strongest recovery response following extract application. Crop-level screening revealed clear interspecific differences in drought sensitivity and responsiveness, supporting the selection of cucumber as a suitable model for detailed physiological and biochemical evaluation. Under drought stress, extract-treated cucumber seedlings exhibited improved leaf water status and photosynthetic performance, and reduced oxidative damage and changes in enzymatic and non-enzymatic antioxidant responses. Integrated indices and heatmap analyses confirmed that these protective effects were maintained under prolonged drought conditions, with *P. guajava* and *A. sativum* generally exhibiting response patterns closer to the control across multiple physiological and biochemical parameters.

These findings suggest that under controlled greenhouse conditions, guava and garlic extracts may be able to reduce drought stress in cucumber seedlings. However, before useful agricultural suggestions can be made, more research is needed to confirm their efficacy at later growth stages and in field settings, as well as to standardize extract composition, application concentration, time, and frequency. The cost-effectiveness and practical feasibility under field conditions remain to be evaluated. Overall, the findings support the continued development and optimization of biostimulant formulations that can be readily integrated into existing crop management practices. Priority areas for future research include: (1) multi-location field trials to validate greenhouse findings under variable environmental conditions, (2) biochemical characterization of extracts to identify active compounds and enable standardization, (3) optimization of application timing and frequency across crop growth stages, (4) economic analysis comparing extract application costs to yield benefits, and (5) investigation of potential synergistic effects when extracts are combined with other stress-management practices.

## Figures and Tables

**Table 1:** Crop-level summary of drought tolerance index based on plant height and shoot fresh weight of different crops at 7 days after treatment.

Crop	Stress DI (PH)	Stress DI (SFW)	Optimal Conc.	PH DI at 0.1%	SFW DI at 0.1%	Response Pattern
Barley	0.78 ^a^	0.41 ^b^	0.1	0.95 ^a^	0.71 ^b^	Moderate recovery
Maize	0.69 ^b^	0.4 ^b^	0.1	0.81 ^c^	0.56 ^c^	Limited recovery, low SFW responsiveness
Rice	0.80 ^a^	0.54 ^a^	0.1	0.90 ^b^	0.67 ^b^	Stable performance, low stress sensitivity
Cucumber	0.68 ^b^	0.39 ^b^	0.1	0.94 ^a^	0.79 ^ab^	Strong recovery in both PH and SFW

Conc., concentration; PH, plant height; SFW, shoot fresh weight; DI, drought tolerance index; Different letters indicate significant differences among crops (Tukey’s HSD, *p* < 0.05).

## Data Availability

The data presented in this study are available within the article and its Supplementary Materials. Additional data are available from the corresponding author upon request.

## Supplementary Materials

The following supporting information can be downloaded at: https://www.mdpi.com/article/10.3390/plants15193055/s1, Table S1: Effects of different concentrations of selected plant extracts on plant height and shoot fresh weight of four crop species under drought stress; Table S2: Absolute antioxidant enzyme activities (U mg^−1^ protein) in cucumber leaves under drought stress at 5 and 7 DAT; Figure S1: Principal component analysis (PCA) biplots of physiological and biochemical variables in cucumber seedlings at 5 DAT (A) and 7 DAT (B). PCA was performed using standardized (z-score transformed) biological-replicate-level data for relative water content (RWC), maximum quantum efficiency of photosystem II (Fv/Fm), total chlorophyll (Chl), carotenoids (Caro), hydrogen peroxide (H₂O₂), malondialdehyde (MDA), superoxide dismutase (SOD), catalase (CAT), ascorbate peroxidase (APX), guaiacol peroxidase (GPOX), ascorbate (AsA), and glutathione (GSH). PC1 and PC2 explained 36.17% and 27.94% of the total variation at 5 DAT and 44.50% and 23.06% at 7 DAT, respectively. Each symbol represents one biological replicate (n = 3). CK, non-stressed control; DS, drought-stressed control; DS+E1, drought + *Psidium guajava* extract; DS+E2, drought + *Aloe vera* extract; DS+E3, drought + *Allium sativum* extract; DS+E4, drought + *Medicago sativa* extract. DAT, days after treatment.
