# Supplementary material for: Crop-Level Screening and Physiological Evaluation Reveal Drought-Protective Effects of Plant-Derived Extracts in Cucumber Seedlings
Source: Plants (Basel). 2026 Oct 7;15(19):3055. doi: 10.3390/plants15193055 (PMC13666855; doi:10.3390/plants15193055)
Supplement: Supplementary file 1 [file plants-15-03055-s001.zip › plants-4499729-supplementary.pdf]

Supplementary Materials

Table S1. Effects of different concentrations of selected plant extracts on plant height and shoot fresh weight of four crop species under drought stress.

Treatment	Concentration (%)	Plant height (cm)				Shoot fresh weight (g)			
		C1	C2	C3	C4	C1	C2	C3	C4
Control	0	20.50^{a} (100)	57.33^{a} (100)	30.88^{a} (100)	26.55^{a} (100)	9.67^{a} (100)	15.00^{a} (100)	4.58^{a} (100)	0.809^{a} (100)
DS	0	14.00^{b} (68)	39.57^{a} (69)	24.23^{b} (78)	21.17^{c} (80)	3.77^{c} (39)	6.00^{b} (40)	1.88^{c} (41)	0.435^{b} (54)
DS+E1	0.05	18.50^{a} (90)	46.67^{a} (81)	28.87^{ab} (93)	23.06^{bc} (87)	7.00^{ab} (72)	9.00^{b} (60)	2.80^{bc} (61)	0.552^{ab} (68)
	0.1	19.33^{a} (94)	49.17^{a} (86)	29.32^{ab} (95)	23.78^{a-c} (90)	8.00^{ab} (83)	9.40^{b} (63)	3.13^{bc} (68)	0.574^{ab} (71)
	0.5	19.27^{a} (94)	49.00^{a} (86)	28.9^{ab} (94)	23.83^{a-c} (90)	7.67^{ab} (79)	9.33^{b} (62)	2.92^{bc} (64)	0.569^{ab} (70)
	1	17.43^{ab} (85)	45.17^{a} (79)	27.83^{ab} (90)	22.73^{bc} (86)	6.67^{b} (69)	8.87^{b} (59)	3.11^{bc} (68)	0.498^{ab} (62)
DS+E2	0.05	17.00^{ab} (83)	45.67^{a} (80)	26.97^{ab} (87)	22.29^{bc} (84)	6.50^{bc} (67)	6.33^{b} (42)	3.09^{bc} (67)	0.496^{ab} (61)
	0.1	18.17^{a} (89)	47.50^{a} (83)	28.89^{ab} (94)	23.61^{a-c} (89)	7.33^{ab} (76)	8.33^{b} (56)	3.49^{ab} (76)	0.543^{ab} (67)
	0.5	17.67^{ab} (86)	47.33^{a} (83)	27.59^{ab} (89)	22.94^{bc} (86)	7.33^{ab} (76)	7.33^{b} (49)	3.19^{bc} (70)	0.545^{ab} (67)
	1	16.67^{ab} (81)	44.67^{a} (78)	27.06^{ab} (88)	23.11^{bc} (87)	6.33^{bc} (65)	5.80^{b} (39)	2.46^{bc} (54)	0.494^{ab} (60)
DS+E3	0.05	18.67^{a} (91)	44.33^{a} (77)	27.60^{ab} (89)	23.23^{bc} (87)	6.67^{b} (69)	6.33^{b} (42)	2.57^{bc} (56)	0.510^{ab} (63)
	0.1	20.17^{a} (98)	49.67^{a} (87)	29.33^{ab} (95)	24.28^{ab} (91)	7.00^{ab} (72)	9.00^{b} (60)	3.34^{ab} (73)	0.540^{ab} (67)
	0.5	18.93^{a} (92)	46.67^{a} (81)	29.00^{ab} (94)	23.83^{a-c} (90)	7.67^{ab} (79)	7.33^{b} (49)	2.97^{bc} (65)	0.557^{ab} (69)
	1	17.00^{ab} (83)	43.00^{a} (75)	26.16^{ab} (85)	23.12^{bc} (87)	6.50^{bc} (67)	6.00^{b} (40)	2.57^{bc} (56)	0.487^{ab} (60)
DS+E4	0.05	17.00^{ab} (83)	46.00^{a} (80)	27.49^{ab} (89)	23.16^{bc} (87)	6.60^{b} (68)	6.40^{b} (43)	2.89^{bc} (63)	0.502^{ab} (62)
	0.1	18.67^{a} (91)	47.50^{a} (83)	29.31^{ab} (95)	23.94^{a-c} (90)	7.33^{ab} (76)	7.00^{b} (47)	3.24^{ab} (71)	0.531^{ab} (66)
	0.5	18.67^{a} (91)	46.33^{a} (81)	29.21^{ab} (95)	23.56^{a-c} (89)	7.50^{ab} (78)	6.70^{b} (45)	2.88^{bc} (63)	0.553^{ab} (68)
	1	17.67^{ab} (86)	45.33^{a} (79)	27.00^{ab} (87)	22.67^{bc} (85)	6.67^{b} (69)	5.67^{b} (38)	2.67^{bc} (58)	0.519^{ab} (64)

Values represent means of three biological replicates (n = 3). Values in parentheses indicate the percentage of the corresponding non-stressed control (Control = 100%). Different lowercase letters within the same column indicate significant differences among treatments according to the LSD test at $P < 0.05$. DS, drought-stressed control; E1, *Psidium guajava* extract; E2, *Aloe vera* extract; E3, *Allium sativum* extract; E4, *Medicago sativa* extract; C1, barley; C2, maize; C3, rice; C4, cucumber.

Table S2. Absolute antioxidant enzyme activities (U mg $^{-1}$ protein) in cucumber leaves under drought stress at 5 and 7 DAT.

Days after treatment (DAT)	Treatments	SOD	CAT	APX	GPX
5 DAT	CK	50.24 ± 4.09^{ab}	0.497 ± 0.030^{b}	10.16 ± 0.12^{d}	0.220 ± 0.005^{c}
	DS	41.30 ± 1.47^{b}	0.510 ± 0.015^{b}	24.67 ± 0.26^{a}	0.357 ± 0.010^{b}
	DS+E1	24.67 ± 1.47^{c}	0.329 ± 0.009^{b}	15.64 ± 0.65^{c}	0.473 ± 0.016^{a}
	DS+E2	53.07 ± 2.15^{a}	0.847 ± 0.040^{a}	19.84 ± 0.83^{b}	0.371 ± 0.004^{b}
	DS+E3	26.30 ± 2.61^{c}	0.776 ± 0.062^{a}	14.61 ± 0.18^{b}	0.464 ± 0.015^{a}
	DS+E4	25.08 ± 2.63^{c}	0.794 ± 0.085^{a}	19.18 ± 0.37^{c}	0.384 ± 0.015^{b}
7 DAT	CK	61.43 ± 6.55^{a}	0.408 ± 0.021^{b}	8.08 ± 0.52^{d}	0.191 ± 0.002^{e}
	DS	75.19 ± 2.19^{a}	0.540 ± 0.036^{ab}	19.82 ± 0.23^{a}	0.291 ± 0.004^{d}
	DS+E1	52.26 ± 2.26^{a}	0.230 ± 0.033^{c}	10.67 ± 0.51^{c}	0.427 ± 0.011^{a}
	DS+E2	73.84 ± 1.85^{a}	0.613 ± 0.046^{a}	17.94 ± 0.46^{ab}	0.344 ± 0.014^{c}
	DS+E3	52.26 ± 2.26^{a}	0.225 ± 0.012^{c}	11.15 ± 0.10^{c}	0.410 ± 0.009^{ab}
	DS+E4	51.23 ± 1.95^{a}	0.418 ± 0.050^{b}	15.79 ± 0.51^{b}	0.367 ± 0.005^{bc}

Values are presented as **mean ± SE** (n = 3 biological replicates). SE was calculated from the standard deviation (SD/√3). These absolute enzyme activity values were used to the relative enzyme activities (control = 1.0), which were subsequently standardized by z-score normalization to generate the heatmap shown in **Figure 6**. Data are presented as mean ± SE of three biological replicates (n = 3).

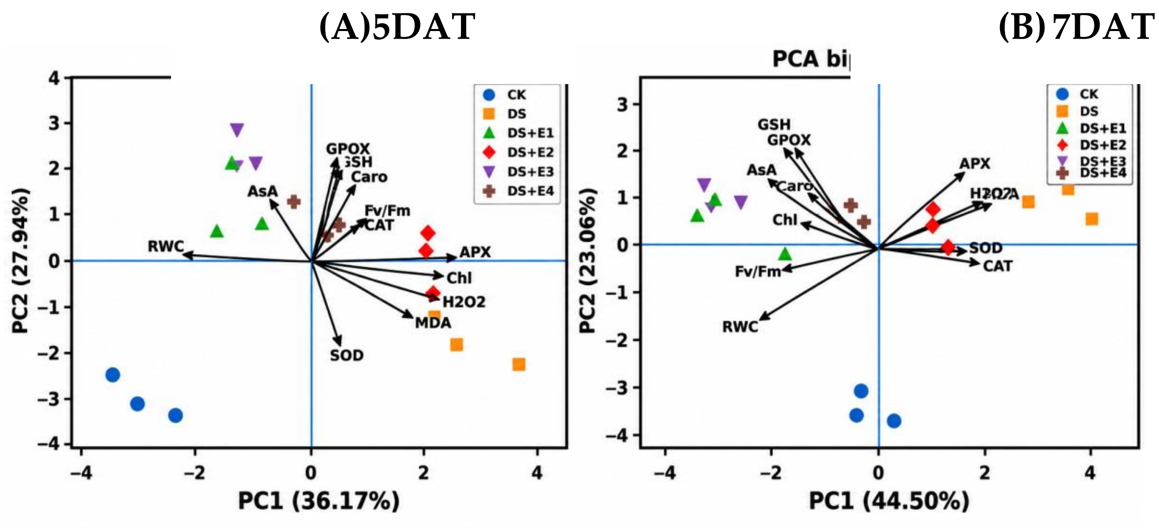

Figure S1. Principal component analysis (PCA) biplots of physiological and biochemical variables in cucumber seedlings at 5 DAT (A) and 7 DAT (B). PCA was performed using standardized (z-score transformed) biological-replicate-level data for relative water content (RWC), maximum quantum efficiency of photosystem II (Fv/Fm), total chlorophyll (Chl), carotenoids (Caro), hydrogen peroxide (H_2O_2), malondialdehyde (MDA), superoxide dismutase (SOD), catalase (CAT), ascorbate peroxidase (APX), guaiacol peroxidase (GPOX), ascorbate (AsA), and glutathione (GSH). PC1 and PC2 explained 36.17% and 27.94% of the total variation at 5 DAT and 44.50% and 23.06% at 7 DAT, respectively. Each symbol represents one biological replicate (n = 3). CK, non-stressed control; DS, drought-stressed control; DS+E1, drought + *Psidium guajava* extract; DS+E2, drought + *Aloe vera* extract; DS+E3, drought + *Allium sativum* extract; DS+E4, drought + *Medicago sativa* extract. DAT, days after treatment.
